# Mysterious Linkages Between Hepatitis C Virus Genotypes, Interleukin-28B Genotypes and Viral Clearance- A Meta-Analysis

**DOI:** 10.5812/hepatmon.15895

**Published:** 2014-03-02

**Authors:** Seyed Hossein Aalaei-Andabili, Bita Behnava, Shima Salimi, Heidar Sharafi, Seyed Moayed Alavian

**Affiliations:** 1 Molecular Immunology Research Center, Department of Immunology, School of Medicine, Tehran University of Medical Sciences, Tehran, IR Iran; 2 Middle East Liver Disease Center, Tehran, IR Iran; 3 Baqiyatallah Research Center for Gastroenterology and Liver Disease (BRCGL), Baqiyatallah University of Medical Sciences, Tehran, IR Iran; 4 Iran Hepatitis Network (IHN), Tehran, IR Iran

**Keywords:** Hepatitis C, IL28B Protein, Human, Meta-Analysis, Genotype

## Abstract

**Background:**

Recent genome wide association studies (GWAS) have shown important roles of single nucleotide polymorphisms (SNP) near region of interleukin B 28 (IL28B) gene in spontaneous and drug-induced clearance of hepatitis C virus (HCV) in genotype 1 HCV infection.

**Objectives:**

This meta-analysis was designed to determine the world-wide distribution patterns of IL28B genotypes and alleles, and to find possible linkages between IL28B and HCV genotypes.

**Patients and Methods:**

Manual and electronic databases were searched. Critical appraisal was performed. According to the results of heterogeneity tests, we used fix/random model for the analysis. The data concerning patients’ ethnicity and HCV genotypes were analyzed by using statistical analysis software.

**Results:**

A total of 255 articles were found. After article review and quality assessment, 50 studies, including 18662 patients and 1313 healthy subjects, were analyzed. Presence of HCV genotype 3 versus genotype 1 was significantly associated with a higher frequency of CC genotype and C allele, with an odds ratio (OR) of 1.68 (95% CI: 1.44-1.99) and 1.49 (95% CI: 1.33-1.67), respectively. Prevalence of the rs12979860 CC genotype among genotype 1 HCV infected patients of Asian ethnicity was 69.48% (95% CI: 65.20-73.77), which was significantly higher than its prevalence [33.27% (95% CI: 28.88-37.67)] in the Caucasian genotype 1 HCV infected patients. Prevalence of rs12979860 TT genotype in the African-American genotype 1 HCV infected patients was the highest [36.20% (95% CI: 32.91-39.49)], and significantly different compared to all other ethnicities.

**Conclusions:**

There were significant linkages between HCV genotypes and IL28B genotypes/alleles. Patients with a favorable IL28B and genotypes 1 and 4 HCV infection stand a better chance to clear HCV in the acute phase.

## 1. Background 

Hepatitis C virus (HCV) has been recognized as a main cause of cirrhosis and hepatocellular carcinoma globally (1). Following an acute infection, 70% to 80% of infected patients fail to clear HCV RNA, and progress to chronic hepatitis C (2). Current standard therapy with pegylated interferon alpha (Peg IFN) and ribavirin has resulted in a 40% to 50% sustained virologic response (SVR) in genotype 1 HCV infected patients (3). The effect of several factors, such as HCV genotype, pretreatment HCV viral load, and stage of liver fibrosis, on the response to antiviral therapy, has been reported. Also, the type of prescribed interferon (2a versus 2b) (4), ribavirin dosage (5), and optimal duration of antiviral therapy have been recognized as other determinant factors of response to treatment (6). It has been seen that response rates are variable in different races. The African-American ethnicity is related with a poor response to antiviral therapy, compared to Caucasians. On the other hand, the Asian ethnicity patients have the highest response rate to the therapy (7).

Recent genome wide association studies (GWAS) have shown that single nucleotide polymorphisms (SNP) near the region of interleukin B 28 (IL28B) gene play an important role in spontaneous clearance of HCV RNA, and response to Peg IFN and ribavirin combination therapy, among patients with genotype 1 HCV infection (8-11). Two SNPs, rs8099917 and rs12980275, strongly associated with virologic response and spontaneous HCV clearance, were found (12). However, data regarding the association of IL28B polymorphisms with viral clearance and treatment response rates among patients with HCV genotype 2 and 3 infection are conflicting (13, 14).

In addition to the ethnicity variation of IL28B polymorphisms, it seems that there is an association between frequency of IL28B genotypes and alleles with HCV genotypes. Two new studies have reported that the IL28B genotypes distribution variation depends on HCV genotypes (15, 16). However, the possible mechanisms capable of justifying this finding are not well understood.

## 2. Objectives

The aim of this study was to confirm the hypothesis regarding the association of IL28B genotypes and alleles with HCV genotypes. We performed a systematic review and meta-analysis of available literature to assess the distribution of IL28B genotypes and alleles in different HCV genotypes, based on patients' ethnicity. In addition, we focused to find possible associations of IL28B genotypes and alleles with HCV genotypes.

## 3. Patients and Methods

### 3.1. Search Strategy

Electronic and manual research of specialty journals and congress books were conducted to find all pertinent literature. We started our electronic search from three MEDLINE database engines (PubMed, EMBASE, and Ovid), Scopus, and ISI. Then, we performed searches on Google Scholar, after finding the last article that complied with our research, we continued the search on Google, up to the moment when we had 200 consecutive links unrelated with our study. In addition, we searched with Google search engine to find gray literature relevant for our study. Nevertheless, we researched for papers presented during congresses, by mentioning “congress” and “conference”, as the keywords. Moreover, backward and forward searches were performed by screening all references (to identify possible studies missed in our search) in related articles. An email was sent to the first or corresponding authors, if the full texts of their articles were not accessible. Data from abstracts were extracted, while excluding non-informative abstracts, if no response was received a month after our email to the authors. We checked the search sensitivity by counting the duplicated articles. We used terms such as “hepatitis C” or “HCV” and “interleukin 28B” or “IL28B” as keywords. We tried to include all published and unpublished studies till the 20th of February 2012.

### 3.2. Quality Assessment of Relevant Studies

Critical appraisal (CA) was performed by using the Epible check (17) list by three investigators (SH AA, SM A, B B). We coordinated meetings before the CA and investigators were justified and trained about questions. Then, the CA was performed for 20 selected articles and a pilot meeting was organized to find the Kappa coefficient between investigators. If there was more than a two numbers difference between CA scores, investigators discussed about, and, if they did not convince, the article was referred to the third person (H SH) for rechecking. The articles were divided to three groups according to CA scores: high quality articles (total CA score > 70%), moderate quality (total CA score between 40%-70%) and, low quality (total CA score < 40%). We decided to exclude all low quality articles from the analysis.

### 3.3. Inclusion and Exclusion Criteria

Published studies in all languages were eligible if they met the following criteria: 1) English, French, and Persian full texts or an informative abstract in English; 2) Appropriate study design: cross-sectional, case-control, clinical trial, or cohort; 3) Studies clearly stated information about patients’ ethnicity, HCV genotype, and IL28B genotype and allele.

 Following items were considered as exclusion criteria: 1) Studies with unclear and confusing data; 2) Studies that have reported results without ethnicity stratification; 3) Studies in which HCV genotypes or IL28B genotypes/alleles were not well defined.

### 3.4. Data Extraction

Data extraction was completed by three investigators (SH. AA, B B, SH S) and then rechecked by one investigator (SH AA). We extracted: first author’s name, publication year, name of country, type of study, number of included patients, patients characteristics, patients ethnicity, HCV genotype, and IL28B genotype and allele, if full text of articles were available. Extracted data were categorized for each ethnicity and HCV genotype in Microsoft Office Excel 2007 (Redmond, Washington, The USA). Informative English abstracts with non-English full texts were referred to a translator for full text checking.

### 3.5. Data Modification and Statistical Analysis

Statistical heterogeneity of results was appraised using heterogeneity tests such as: Q-Squared, I-Squared, and Tau statistic. For Q statistics, a P value < 0.1 was considered as significant. I-squared lies between 0% and 100% and heterogeneity increases with increasing of I-squared value. Since there were few articles in several subgroups, we used Tau statistic because it is not influenced by the number of included studies (18). According to the results of heterogeneity tests, we used the fix/random model (DerSimonian and Laird) for our meta-analysis method with “metan” command. Subgroup analysis was performed if at least three eligible studies were available (19). Difference of IL28B genotypes and alleles prevalence between different HCV genotypes or patients ethnicity were considered significant, if the confidence intervals (95% CI) of the prevalence were not over-lapped. Also, the odds ratios (OR) and its 95% CI were calculated to determine HCV genotypes and IL28B genotypes/alleles linkages using the Mantel Hansel approach. The analysis was performed by using the STATA 11 software (STATA Corp. LP, Texas, The USA).

## 4. Results

### 4.1. Search Result

Two hundred thirty two articles were found in our online literature review. Twenty three other articles were added after the manual search of journals and congress books. Forty eight review articles and 11 letters to editors were excluded. In addition, 12 papers were duplicated, and three studies had repeated the same data of multicentric studies in other styles. Further, five articles including HIV-HCV co-infected patients were excluded alongside another 126 articles that did not clearly state HCV genotype, patients’ ethnicity, or IL28B genotype/allele.

**Figure 1.**
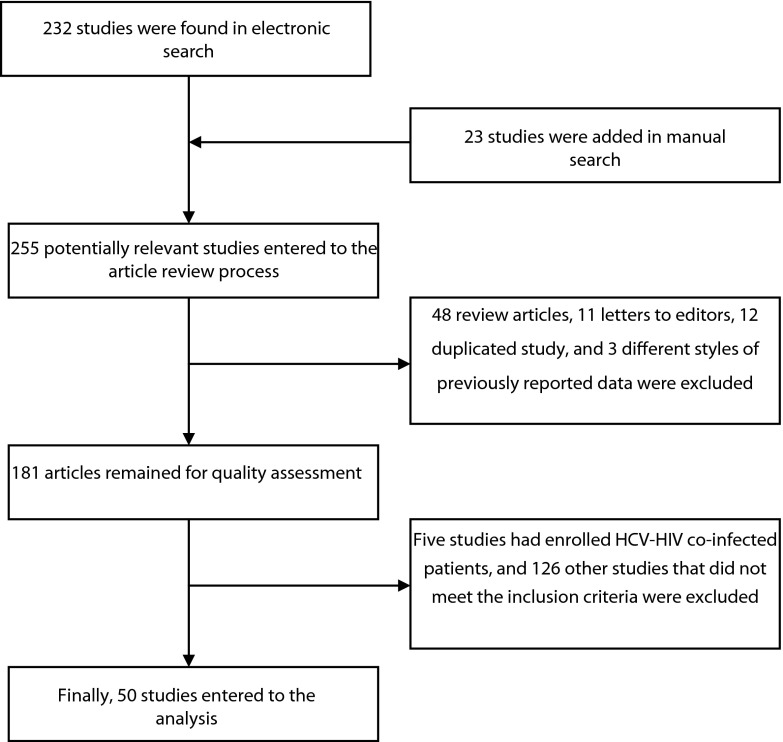
Flow Diagram of Search and Study Selection

Finally, 50 articles, including 18662 patients and 1313 healthy subjects, entered the final analysis. Figure 1 reveals the flow diagram of search. Included articles are shown in supplementary information 1.

The agreement between authors for study selection was good (Kappa coefficient: 0.85). All articles were published between 2009 and 2012. 

Twenty-nine studies were found regarding the Caucasian patients. In this ethnicity, 10089 patients (including 8652 genotype 1, 369 genotype 2, 770 genotype 3, and 298 genotype 4 cases) and 1313 healthy subjects were involved. The mean age of the patients ranged from 24.6 to 53 years. Two studies enrolled females only. In other reports, most of the study subjects were males (ranging from: 52.5% - 91.2%).

Patients with genotype 3 HCV infection had the highest rate of IL28B rs12979860 CC genotype in the Caucasian population, compared to subjects with genotype 4 infection who scored for the lowest rate. The CC genotype prevalence among patients with genotype 3 [48.35% (95% CI: 42.14-54.56)] was significantly higher than its prevalence among genotype 1 [33.27% (95% CI: 28.88-37.67) and genotype 4 [28.41% (95% CI: 23.32-33.51)] patients. Also, CC genotype prevalence among genotype 2 HCV infected patients [41.84% (95% CI: 36.84-46.83)] was significantly higher than in patients with genotype 4 HCV infection. In addition, the prevalence of rs8099917 TT genotype among patients with genotype 3 HCV infection [72.58% (95% CI: 68.47-76.69)] was significantly higher than for genotype 1 HCV infected patients [53.18% (95% CI: 49.41-56.96)]. Due to lack of published studies, the pooled estimation of rs8099917 genotypes was not possible among subjects belonging to genotypes 2 and 4 (Table 1). 

**Table 1.** Prevalence of IL28B Genotypes and Alleles Among Different Hepatitis C Virus Genotypes in Caucasian, Asian, and African-American Ethnicity

**Caucasian, %** (**mean**)										
**1**	33.27 (28.88-37.67)	50.70 (47.91-53.50)	14.45 (12.82-16.08)	5.60 (4.33-6.86)	40.70 (38.25-43.15)	53.18 (49.41-56.96)	59.59 (57.45-61.73)	40.41 (38.27-42.55)	26.35 (23.86-28.83)	73.65 (71.17-76.14)
**2**	41.84 (36.84-46.83)	46.34 (41.29-51.39)	11.30 (8.08-14.53)	-	-	-	65.57 (61.78-69.37)	34.43 (30.63-38.22)	-	-
**3**	48.35 (42.14-54.56)	45.13 (40.47-49.79)	6.19 (3.32-9.05)	3.36 (1.49-5.23)	25.52 (20.50-29.54)	72.58 (68.47-76.69)	71.21 (66.73-75.68)	28.79 (24.32-33.27)	14.52 (12.22-16.81)	85.48 (83.19-87.78)
**4**	28.41 (23.32-33.51)	57.15 (51.56-62.74)	13.80 (9.89-17.71)	-	-	-	57.42 (53.45-61.38)	42.58 (38.62-46.55)	-	-
**Healthy Subjects**	45.44 (41.55- 49.33)	42.32 (40.08- 44.71)	10.98 (9.51- 12.45)	-	-	-	67.54 (65.98- 69.09)	32.48 (30.92- 34.03)	-	-
**Asian, %** (**mean**)										
**1**	69.48 (65.20-73.77)	27.49 (23.59-31.40)	2.89 (1.71-4.06)	1.73 (1.39-2.06)	26.92 (24.46-29.38)	71.13 (68.68-73.75)	83.51 (80.94-86.09)	16.49 (13.91-19.06)	15.13 (13.94-16.33)	84.87 (83.67-86.06)
**2**	-	-	-	1.31 (0.73-1.89)	18.78 (16.83-20.74)	79.72 (77.71-81.73)	-	-	10.85 (9.56-12.15)	89.15 (87.85-90.44)
**African-American, % **(**mean**)										
**1**	13.07 (10.76-15.37)	50.20 (46.79-53.61)	36.20 (32.91-39.49)				38.56 (36.20-40.92)	61.44 (59.08-63.80)	-	-

C allele was the most prevalent allele in genotype 3 HCV infected patients [71.21% (95% CI: 66.73-75.68)] in allele analysis, followed by patients with genotype 2 who had a higher rate of C allele [65.57% (95% CI: 61.78-69.37)] compared to others. Prevalence of C allele among patients with genotypes 1 [59.59% (95% CI: 57.45-61.73)] and 4 [57.42% (95% CI: 53.45-61.38)] accounted for no significant different. Prevalence of C allele among genotype 3 patients was significantly higher than its prevalence among patients with genotypes 1 and 4 HCV infection. In addition, patients with genotype 2 had significantly higher levels of the C allele versus patients with genotypes 1 and 4 HCV infection. The prevalence of rs8099917 T allele in genotype 3 patients [85.48% (95% CI: 83.19-87.78)] was also significantly higher than its prevalence [73.65% (95% CI: 71.17-76.14)] in patients with genotype 1.

**Figure 2.**
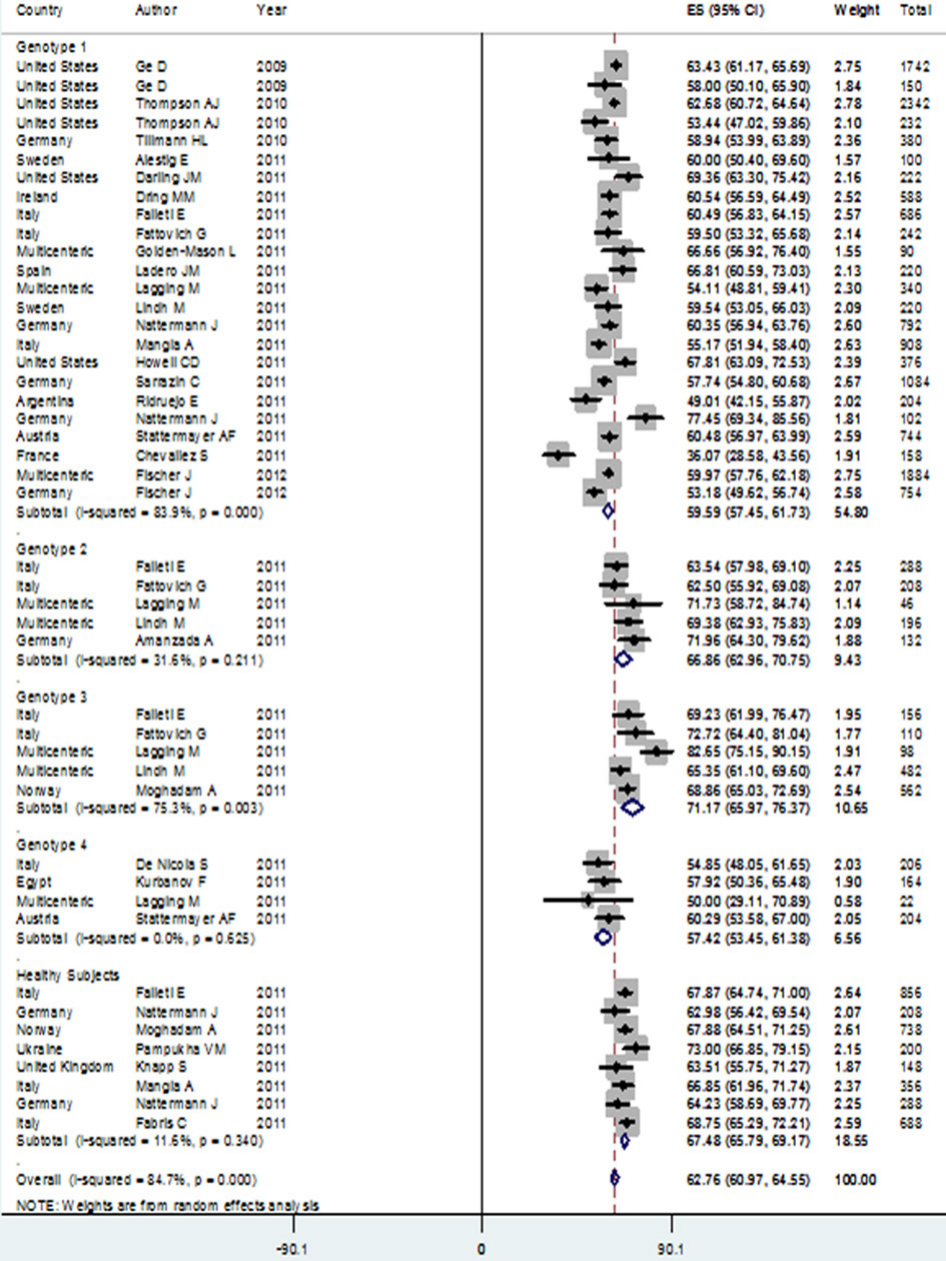
C Allele Distribution in Different Hepatitis C Virus Genotype Patients and Healthy Controls in Caucasian Ethnicity

We found that rs12979860 CC genotype and C allele prevalences among healthy subjects were similar to their distributions in genotypes 2 and 3 HCV infected patients, yet they significantly differed from rs12979860 CC genotype and C allele distributions in genotypes 1 and 4 HCV infected patients (Figure 2). 

Interestingly, we found that the presence of HCV genotype 3 compared to HCV genotype 1 was significantly associated with a higher level of CC genotype and C allele, with the ORs of 1.68 (95% CI: 1.44-1.99) and 1.49 (95% CI: 1.33-1.67), respectively. The highest HCV genotype linkage with IL28B CC genotype and C allele was seen in genotype 3 HCV infection versus genotype 4, with ORs of 2.13 (95% CI: 1.59-2.84) and 1.67 (95% CI: 1.37-2.03), respectively.

In addition, genotype 2 HCV infection versus genotype 1 was significantly associated with an increased presence of the C allele [OR: 1.25 (95% CI: 1.07-1.46)].

The HCV infection with genotype 3 compared to genotype 1 was significantly associated with higher rs8099917 TT genotype [OR: 2.31 (95% CI: 1.85-2.87)], and presence of T allele [OR: 2.07 (95% CI: 1.50-2.51)] (Table 2). 

**Table 2.** Hepatitis C Virus Genotypes Effects on the Presence of IL28B Favorable Genotypes and Alleles

**Caucasian**			
**C allele**	genotype 3 vs. genotype 1	1.49	1.33 – 1.67
**C allele**	genotype 3 vs. genotype 2	1.19	0.99 – 1.43
**C allele**	genotype 3 vs. genotype 4	1.67	1.37 – 2.03
**C allele**	genotype 2 vs. genotype 1	1.25	1.07 – 1.46
**CC**	genotype 3 vs. genotype 1	1.68	1.44 – 1.95
**CC**	genotype 3 vs. genotype 2	1.19	0.92 – 1.53
**CC**	genotype 3 vs. genotype 4	2.13	1.59 – 2.84
**CC**	genotype 2 vs. genotype 1	1.13	0.93 – 1.40
**T allele of rs80**	genotype 3 vs. genotype 1	2.07	1.70 – 2.51
**TT of rs80**	genotype 3 vs. genotype 1	2.31	1.85 – 2.87
**Asian**			
**T allele of rs80**	genotype 2 vs. genotype 1	1.43	1.26 – 1.62
**TT of rs80**	genotype 2 vs. genotype 1	1.50	1.31 – 1.73

### 4.2. Asian 

Twenty one studies were included into the final analysis [supplementary information 1]. In the Asian race, 7755 (6220 genotype 1 and 1535 genotype 2) HCV infected patients were identified. No report regarding genotypes 3 and 4 patients was eligible. Patients' mean age ranged from 52.4 to 64 years. One study included more females (50.38% versus 49.62%). Other studies predominantly enrolled male gender patients with a proportion ranging from 50.4% to 75%. 

**Figure 3.**
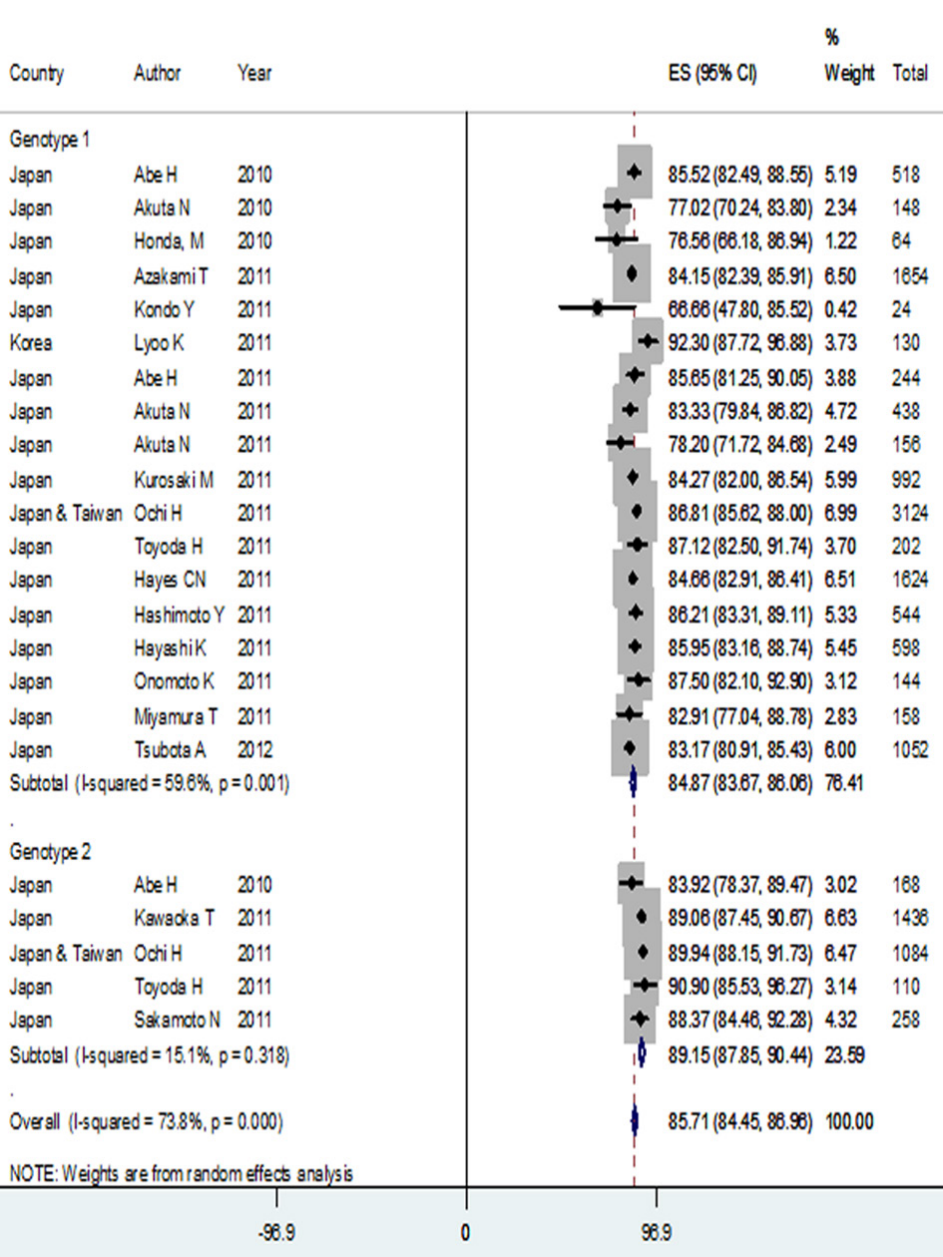
T Allele Prevalence Among Genotypes 1 and 2 HCV Infected Patients of Asian Ethnicity

Prevalence of rs12979860 CC genotype among genotype 1 HCV infected patients in the Asian ethnicity was 69.48% (95% CI: 65.20-73.77), which was significantly higher than its prevalence among genotype 1 HCV infected patients in the Caucasians. In contrast, rs12979860 TT genotype rate was significantly higher among the Caucasian patients [14.45 % (95% CI: 12.82-16.08)] compared to Asians [2.89 % (95% CI: 1.71-4.06)] in genotype 1 HCV infection. In rs8099917 comparison, the prevalence of TT genotype in genotype 1 HCV infected patients was 71.13 % (95% CI: 68.68-73.75), which is significantly lower than the TT prevalence in Asian genotype 2 HCV infected patients [79.72% (95% CI: 77.71-81.73)]. The prevalence of rs8099917 TT genotype in genotype 1 HCV infected patients of Asian race was significantly higher than the TT prevalence in genotype 1 HCV infected Caucasians. Also, similar results were found in allele analysis. Prevalence of rs12979860 C allele among patients with genotype 1 HCV infection was 83.51 % (95% CI: 80.94-86.09), which was significantly higher than C allele prevalence in genotype 1 HCV infected patients of Caucasian ethnicity. Moreover, rs8099917 T allele was significantly more prevalent among subjects with genotype 1 HCV infection in the Asian ethnicity [84.87% (95 %CI: 83.67-86.06)] versus the Caucasians [73.65% (95% CI: 71.17-76.14)] (Table 1 and Figure 3). 

Hepatitis C virus infection with genotype 2 versus genotype 1 was associated with a higher presence of rs8099917 TT genotype and T allele, with ORs of 1.50 (95% CI: 1.31-1.73) and 1.43 (95% CI: 1.26-1.62), respectively. There was not enough data to compare rs12979860 genotypes and alleles between different genotypes of HCV infection in the Asian ethnicity (Table 2). 

### 4.3. African-American

Five studies were found regarding IL28B genotypes distribution in the African-American ethnicity [supplementary information 1]. In this race, 818 genotype 1 HCV infected subjects were identified. Patients' mean age varied between 49 and 51 years. Most subjects were males (ranging from 57.33% to 64.20%).

**Figure 4.**
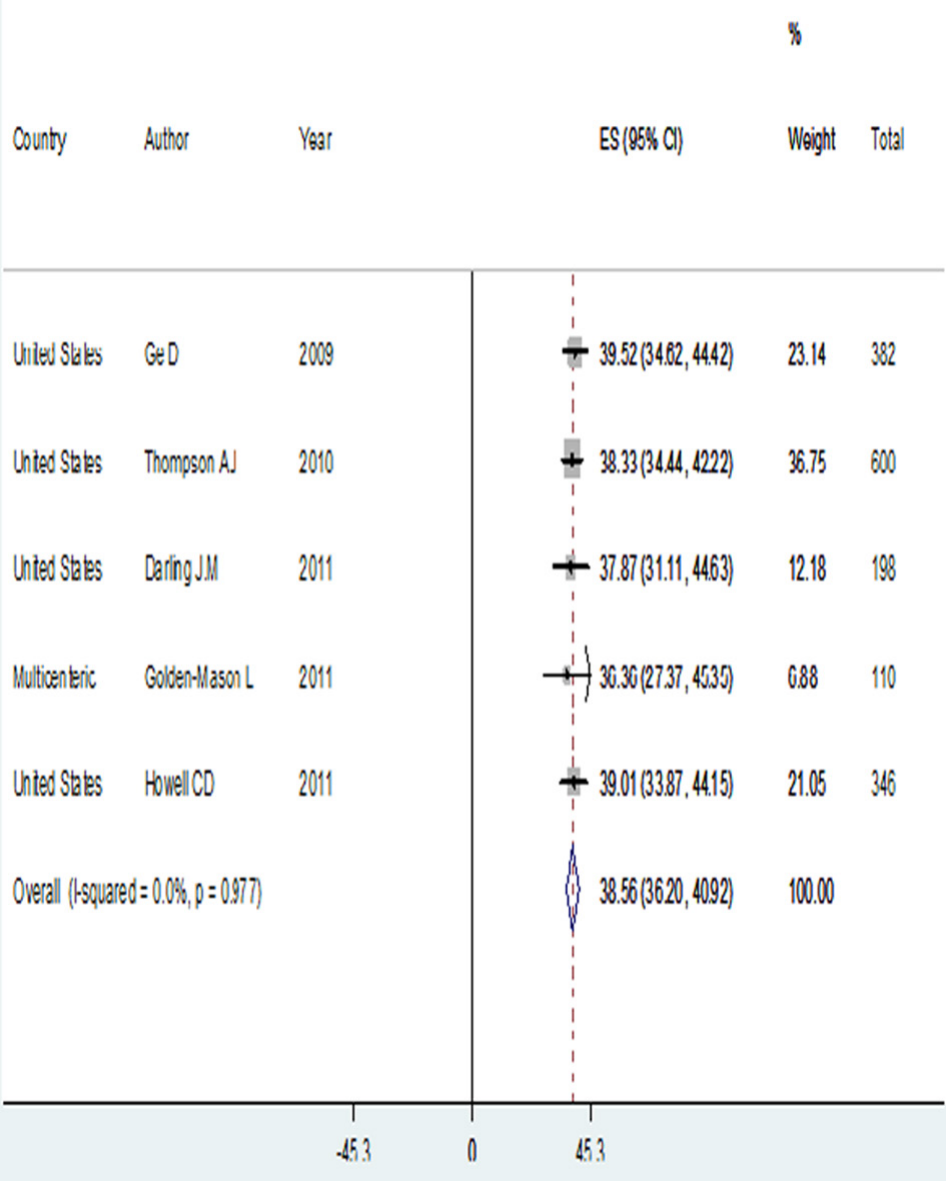
C allele Prevalence Among Genotype 1 Hepatitis C Virus Infected African-American Ethnicity

The prevalence of rs12979860 CC genotype had the lowest rate in this ethnicity, with 13.07% (95% CI: 10.76-15.37) compared to the other races. On other hand, prevalence of rs12979860 TT genotype was the highest, accounting for 36.20% (95% CI: 32.91-39.49), which was significantly different from the other ethnicities. In addition, the prevalence of rs12979860 C allele was 38.56% (95% CI: 36.20-40.92), significantly lower than C allele prevalence among genotype 1 HCV infected patients in the Asian and Caucasian races (Table 1 and Figure 4). 

## 5. Discussion

The treatment of HCV has remained a problem among.

In this meta-analysis, we found that the interaction of HCV genotypes with IL28B rs12979860 and rs8099917 genotypes is not random. Significant associations between favorable HCV genotypes (genotypes 2 and 3) and favorable genotypes of IL28B (rs12979860 CC and rs8099917 TT) were found. Also, higher C allele expressions for rs12979860 and T allele for rs8099917 were seen among favorable genotypes versus the unfavorable. These differences between genotypes justify a higher response to treatment among favorable genotypes of HCV compared to others.

The distributions IL28B rs12979860 and rs8099917 genotypes vary according to patients’ race. We found that Asian HCV infected patients had a higher rate of rs12979860 CC genotype, C allele, rs8099917 TT genotype, and T allele compared to the other ethnicities within viral genotypes. In addition, the prevalence of rs12979860 CC genotype and C allele among patients with unfavorable HCV genotype infection (genotype 1) in the Asian ethnicity were significantly higher than the Caucasian patients with favorable HCV genotype (genotype 3) infection. Although, due to lack of required studies, we could not compare IL28B genotypes and alleles distribution between favorable HCV genotypes of different races; while, the prevalence of favorable genotypes and alleles of both rs8099917 and rs12979860 were significantly higher in the Asian race, compared to the Caucasians and African-Americans. This finding may indicate rs8099917 TT genotype and T allele importance in response to treatment of HCV genotypes 2 and 3 infected patients among Asian subjects.

In this study, HCV infected patients of the African-American ethnicity had the lowest rate of CC genotype between the genotype 1 HCV infected patients, compared to the other races. In addition, this race was the only one with significantly higher levels of rs12979860 CT, TT genotypes, and T allele compared to CC genotype and C allele. This fact shows why patients in the African-American race have the lowest rate of response to therapy. Meanwhile, this rate is the highest among Asian patients. In addition, African-American patients were at a higher risk of liver fibrosis (16). It seems that the lower viral clearance rate among this ethnicity versus others may result from their restricted and smaller region of IL28B, which is associated with HCV clearance (25).

We found that IL28B rs12979860 genotypes and alleles distribution in healthy subjects are significantly different with genotypes 1 and 4 HCV infected patients of Caucasian ethnicity, similar to genotypes 2 and 3 HCV infected patients IL28B rs12979860 genotypes and alleles rates. In addition, significantly lower IL28B rs12979860 CC genotype and C allele rates among HCV genotypes 1 and 4 patients were found, compared to genotypes 2 and 3 subjects. It seems that genotypes 1 and 4 HCV infected patients with a higher rate of rs12979860 CC genotype and C allele could better eradicate HCV RNA in the acute phase of HCV infection, versus genotypes 2 and 3. This ability leads to a lower rate of rs12979860 CC prevalence in the chronic phase of HCV infected patients, compared to genotypes 2 and 3. Moreover, patients progressed to chronic HCV infection had a lower rate of rs12979860 CC genotype.

We think that the host immune system may select the HCV genotype. We would like to hypothesize that the presence of the C allele is associated with a higher rate of viral clearance in both genotypes 4 and 1, versus genotypes 2 and 3. In contrast, it is hypothesized that T cell–specific immune response is associated with IL28B rs12979860 T allele (26). Therefore, T cell–inducing therapeutic vaccine could be more effective among patients with rs12979860 CT and especially, TT genotypes.

The association of liver steatosis (27) and increased jaundice with favorable ILB28 genotype (CC) has been reported (26). We would like to suggest immediate treatment for HCV infected patients of the African-American ethnicity, because of their lower rate of favorable ILB28 genotypes and alleles and their increased risk of asymptomatic disease progression. Concerning Caucasian patients, it is recommended to test for IL28B genotype. Afterwards, the treatment plan should be selected according to the IL28B genotype. In contrast, for Asian patients, it would be valuable to follow the patients before administration of antiviral therapy. This approach prevents drug side effects and is cost-efficient.

Further original studies investigating the acute phase of HCV infection are required. Comparison of IL28B genotypes and alleles distribution in the acute phase of HCV infection, compared to the chronic phase, can show an exact trend of viral clearance among different genotypes of HCV infection.

We were unable to identify any significant limitation in our study.

In conclusion, there were significant linkages between HCV genotypes and IL28B genotypes /alleles. Patients with favorable IL28B and genotypes 1 and 4 HCV infection have a higher chance to clear HCV RNA in the acute phase.
