# Corrigendum to: Mysterious Linkages Between Hepatitis C Virus Genotypes, Interleukin-28B Genotypes and Viral Clearance-A Meta-Analysis [Published in Hepatitis Monthly. 2014 Mar; 14(3): e15895]

**DOI:** 10.5812/hepatmon.22648

**Published:** 2014-09-01

This corrigendum/addendum supplies corrected Table 1 which had missed rows for SNPs and genotypes/alleles.

**Table 1.** Prevalence of IL28B Genotypes and Alleles Among Different Hepatitis C Virus Genotypes in Caucasian, Asian, and African-American Ethnicity ^a^

	rs12979860			rs8099917			rs12979860		rs8099917	
	CC	CT	TT	GG	GT	TT	C	T	G	T
**Caucasian**										
HCV Genotype 1	33.27 (28.88-37.67)	50.70 (47.91-53.50)	14.45 (12.82-16.08)	5.60 (4.33-6.86)	40.70 (38.25-43.15)	53.18 (49.41-56.96)	59.59 (57.45-61.73)	40.41 (38.27-42.55)	26.35 (23.86-28.83)	73.65 (71.17-76.14)
HCV Genotype 2	41.84 (36.84-46.83)	46.34 (41.29-51.39)	11.30 (8.08-14.53)	-	-	-	65.57 (61.78-69.37)	34.43 (30.63-38.22)	-	-
HCV Genotype 3	48.35 (42.14-54.56)	45.13 (40.47-49.79)	6.19 (3.32-9.05)	3.36 (1.49-5.23)	25.52 (20.50-29.54)	72.58 (68.47-76.69)	71.21 (66.73-75.68)	28.79 (24.32-33.27)	14.52 (12.22-16.81)	85.48 (83.19-87.78)
HCV Genotype 4	28.41 (23.32-33.51)	57.15 (51.56-62.74)	13.80 (9.89-17.71)	-	-	-	57.42 (53.45-61.38)	42.58 (38.62-46.55)	-	-
Healthy Subjects	45.44 (41.55-49.33)	42.32 (40.08-44.71)	10.98 (9.51-12.45)	-	-	-	67.54 (65.98-69.09)	32.48 (30.92-34.03)	-	-
**Asian**										
HCV Genotype 1	69.48 (65.20-73.77)	27.49 (23.59-31.40)	2.89 (1.71-4.06)	1.73 (1.39-2.06)	26.92 (24.46-29.38)	71.13 (68.68-73.75)	83.51 (80.94-86.09)	16.49 (13.91-19.06)	15.13 (13.94-16.33)	84.87 (83.67-86.06)
HCV Genotype 2	-	-	-	1.31 (0.73-1.89)	18.78 (16.83-20.74)	79.72 (77.71-81.73)	-	-	10.85 (9.56-12.15)	89.15 (87.85-90.44)
**African-American**										
HCV Genotype 1	13.07 (10.76-15.37)	50.20 (46.79-53.61)	36.20 (32.91-39.49)	-	-	-	38.56 (36.20-40.92)	61.44 (59.08-63.80)	-	-

^a^ Data are presented as mean (95%CI), %.

Corresponding author:

Dr. Seyed Moayed Alavian

